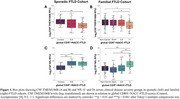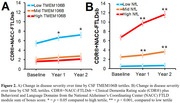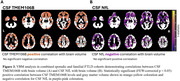# Clinical associations of CSF TMEM106B in familial and sporadic frontotemporal lobar degeneration

**DOI:** 10.1002/alz70856_103985

**Published:** 2025-12-26

**Authors:** Molly Olzinski, Yann Cobigo, Rowan Saloner, Binita Rajbanshi, Julia D Webb, Jingyao Li, Joseph Loureiro, Katie Worringer, Hilary W. Heuer, Peter A. Ljubenkov, Lawren VandeVrede, Adam M. Staffaroni, Argentina Lario Lago, Mark E. Sanderson‐Cimino, Eden V. Barragan, Eliana Marisa Ramos, Casey N. Cook, Leonard Petrucelli, Rosa Rademakers, Brad F. Boeve, Howard J. Rosen, Julio C. Rojas, Adam L. Boxer

**Affiliations:** ^1^ Memory and Aging Center, UCSF Weill Institute for Neurosciences, University of California, San Francisco, San Francisco, CA, USA; ^2^ Weill Institute for Neurosciences, University of California, San Francisco (UCSF), San Francisco, CA, USA; ^3^ Memory and Aging Center, Weill Institute for Neurosciences, University of California San Francisco, San Francisco, CA, USA, San Francisco, CA, USA; ^4^ Novartis Institutes for Biomedical Research, Cambridge, MA, USA; ^5^ Memory and Aging Center, Weill Institute for Neurosciences, University of California, San Francisco, San Francisco, CA, USA; ^6^ Memory and Aging Center, UCSF Weill Institute for Neurosciences, University of California San Francisco, San Francisco, CA, USA; ^7^ Memory and Aging Center, Weill Institute for Neurosciences, University of California San Francisco, San Francisco, CA, USA; ^8^ University of California, San Francisco, San Francisco, CA, USA; ^9^ David Geffen School of Medicine, University of California, Los Angeles, Los Angeles, CA, USA; ^10^ Mayo Clinic, Jacksonville, FL, USA; ^11^ VIB‐UAntwerp Center for Molecular Neurology, University of Antwerp, Antwerp, Antwerp, Belgium; ^12^ Department of Neurology, Mayo Clinic, Rochester, MN, USA; ^13^ UCSF Weill Institute for Neurosciences, University of California, San Francisco, CA, USA; ^14^ Memory and Aging Center, Department of Neurology, Weill Institute for Neurosciences, University of California, San Francisco, San Francisco, CA, USA

## Abstract

**Background:**

*TMEM106B* encodes a lysosomal protein and is a genetic susceptibility factor for frontotemporal lobar degeneration (FTLD). Aptamer‐based proteomics quantifies TMEM106B protein levels in CSF. The associations of CSF TMEM106B with measures of disease severity have not been previously evaluated, and the value of CSF TMEM106B as a clinical biomarker is unknown.

**Method:**

CSF TMEM106B and NfL protein levels were quantified with aptamer‐based proteomics in participants from two independent cohorts: sporadic FTLD with neuropathology‐confirmed FTLD‐tau and FTLD‐TDP, clinically diagnosed PSP‐Richardson Syndrome, and controls (UCSF MAC, n = 95; mean age = 69 ± 6 yrs; 48% female) and familial FTLD comprised of presymptomatic and symptomatic FTLD mutation carriers with family non‐carrier controls (ALLFTD, n = 179; mean age = 49 ± 13 yrs; 53% female). The association between biomarkers and diverse measures of disease severity, including the CDR®+NACC‐FTLD and CDR®+NACC‐FTLD sum of boxes, were determined with linear regressions or mixed linear models. Associations with brain volumes were determined within the framework of voxel‐based morphometry.

**Result:**

In both cohorts, at baseline, CSF TMEM106B did not differ by sex, age, FTLD phenotype, or CSF NfL. CSF TMEM106B was lower in cases with worse disease severity (Figure 1) and in participants with the protective *TMEM106B* rs1990622 G/G genotype, regardless of pathological diagnosis or FTLD‐causing mutation. Low CSF TMEM106B was associated with more severe longitudinal worsening in FTLD disease severity (Figure 2), basic and instrumental function, global cognition, executive function, and depression scores. These associations were weaker than those of NfL. Low CSF TMEM106B was associated with low brain volumes in frontal, parietal, and temporal regions (FWE‐corrected *p* < 0.05) (Figure 3A). CSF NfL negatively correlated (FWE‐corrected *p* < 0.05) with brain volumes in frontotemporal regions (Figure 3B).

**Conclusion:**

CSF TMEM106B concentrations are influenced by the *TMEM106B* rs1990622 genotype, and correlate with disease severity and brain volume in both genetic and sporadic FTLD. Lower levels of CSF TMEM106B correlate with more severe disease and brain atrophy. Disease severity is more strongly associated with CSF NfL, while brain volumes are more strongly associated with CSF TMEM106B. CSF TMEM106B may have value as a biomarker of neurodegeneration in FTLD.